# Amblyopia Treatment through Immersive Virtual Reality: A Preliminary Experience in Anisometropic Children

**DOI:** 10.3390/vision7020042

**Published:** 2023-05-19

**Authors:** Ainhoa Molina-Martín, Luis Leal-Vega, Dolores de Fez, Elena Martínez-Plaza, María Begoña Coco-Martín, David P. Piñero

**Affiliations:** 1 Group of Optics and Visual Perception. Department of Optics, Pharmacology and Anatomy, University of Alicante, 03690 San Vicente del Raspeig, Spain; ainhoa.molina@ua.es (A.M.-M.);; 2 Department of Medicine, Dermatology and Toxicology, Faculty of Medicine, University of Valladolid, 47003 Valladolid, Spain; 3 University of Valladolid, 47003 Valladolid, Spain; 4 Department of Ophthalmology, Vithas Medimar International Hospital, 03016 Alicante, Spain

**Keywords:** virtual reality, perceptual learning, amblyopia, anisometropic, children, vision therapy, dichoptic training

## Abstract

The use of digital devices provides a wide range of possibilities for measuring and improving visual function, including concepts such as perceptual learning and dichoptic therapy. Different technologies can be used to apply these concepts, including, in recent years, the introduction of virtual reality (VR) systems. A preliminary experience in treating anisometropic amblyopia through an immersive VR device and using prototype software is described. A total of 4 children were treated by performing 18 office-based sessions. Results showed that distance VA in amblyopic eyes remained constant in two subjects, whereas the younger subjects improved after the training. Near VA improved in three subjects. All subjects showed an increase in the stereopsis of at least one step, with three subjects showing a final stereopsis of a 60 s arc. A total of three subjects showed an increase of approximately 0.5 CS units for the spatial frequency of 3 cpd after the training. Results from this pilot study suggest that visual training based on perceptual learning through an immersive VR environment could be a viable treatment for improving CS, VA, and stereopsis in some children with anisometropic amblyopia. Future studies should support these preliminary results.

## 1. Introduction

Binocular vision training using video games has become an increasingly popular option for clinicians to treat subjects with binocular abnormalities [1,2], using different options commercially available for this purpose [3,4,5]. The main characteristic of most of these serious games is the use of a dichoptic environment, in which each eye is stimulated with complementary images that would lead to a simultaneous vision of the whole scene as long as fusion exists. Even when suppression is present, as in the case of amblyopia, the problem can be solved by providing the amblyopic eye a visual advantage on the scene [6,7,8] through the use of some stimuli seen only by the amblyopic eye or degrading the luminance of the fellow eye to provide a balanced binocular viewing. In contrast to patching, in which the objective is to penalize the dominant eye and to stimulate the amblyopic eye passively, the binocular treatment has the objective of balancing both eyes, leading to simultaneous vision and, consequently, to binocularity [9]. These binocular treatments are an adjuvant of patching therapies, with improvements in visual acuity when combining both active and passive treatments [10,11,12]. Some previous studies have demonstrated that the effectiveness of the binocular option can be comparable to that obtained with patching [13].

In the case of virtual reality (VR), the mechanism is purely dichoptic, as different and complementary images are seen by each eye (the same scene with a disparity) in different windows, leading to the perception of depth in the virtual space when combined binocularly. Therefore, binocular vision is mandatory to have a fully immersive experience in a virtual reality environment. For this reason, VR devices have been proposed as potential tools for the training of binocular vision in amblyopia [14], specifically for the training of stereoscopic vision [15]. Some authors have previously studied the improvement of stereoscopic vision after VR treatment in adults with anisometropic amblyopia [16,17], using, in some cases, functional magnetic resonance to observe the real neural impact [18], and also in stereoblind subjects [19,20]. This is possibly due to the plasticity of the adult visual system. Likewise, the VR potential as an option for amblyopia treatment in children is not a question of the future, as there are already some recent reports [21,22,23] demonstrating its usefulness.

Since not all VR environments and games are adapted to children with amblyopia, the use of validated software with the use of specific stimuli and effective control over suppression is necessary to provide a clinically significant improvement [24]. For this purpose, the present pilot study aimed to evaluate visual performance changes after an active vision therapy training program in children with anisometric amblyopia using a prototype of an immersive perceptual learning VR-based system.

## 2. Materials and Methods

### 2.1. Subjects

Children were recruited from local health services and evaluated in the Optometric Clinic of the University of Alicante (Alicante, Spain) between September and November 2022. The study protocol was approved by the Drug Research Ethics Committee of the Clinic University Hospital of Valladolid (CASVE-NM361 21-516) and registered in Clinicaltrials.gov (NCT04819386). Informed consent was obtained from the parents or guardians of each child prior to their inclusion, following the Helsinki Declaration tenets.

Inclusion criteria were an age of five years old or over, presence of unilateral amblyopia defined as at least 0.1 logMAR difference in visual acuity (VA) between the eyes (one line), failure of previous passive treatments, such as occlusion, and discontinuation of previous amblyopic treatments at least six weeks prior to the inclusion in the current study. Only children with anisometric amblyopia were considered, defined as amblyopia associated with a difference in objective refraction between eyes of at least one diopter (D) in the spherical equivalent error or 1.50 D in the case of astigmatism. Additionally, subjects with small angle strabismus associated (less than 10 prism diopters) and some grades of global stereopsis were also included. Exclusion criteria were the presence of any class of pathological findings, nystagmus, and/or strabismus over 10 prism diopters (PD).

### 2.2. Training Software

**Figure 1:**
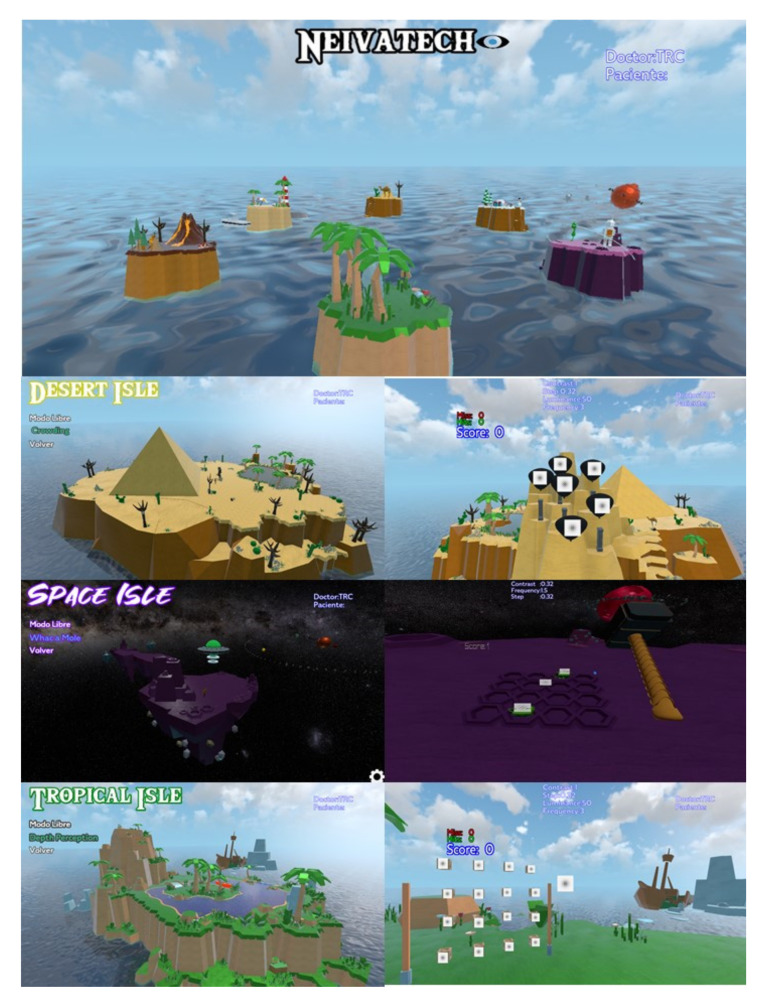
Main scene and mini-games of the prototype of software used for training purposes.

A visual training program based on perceptual learning based on the use of Gabor patches stimulus and specifically designed for a virtual reality environment was used (NEIVATECH, Grupalia Internet SA, Madrid, Spain). The software consists of six thematic islands, with six mini-games on each island in which the subjects develop different tasks. Three of these mini-games were selected for training purposes in the current pilot study: “Crowding” in the Desert Isle, “Whack a Mole” in the Space Isle, and “Depth Perception” in the Tropical Isle (Figure 1). In all mini-games, the Gabor patches stimuli were used during the task. In the first mini-game, the task was to detect the balloons with the target stimulus (a specific Gabor patch) and shoot them from a distance with a laser pointer. In the second mini-game, the task was to punch those Martians emerging in a few seconds from small caves on the floor, using the control, showing a stimulus similar to a specific target (a specific Gabor patch), which required the movement of the subject over the space from a near to intermediate distance. In the third mini-game, the task was to explode wood boxes containing a target stimulus. In contrast to the game with balloons, the task was to explode the boxes guided by stereoscopic cues, i.e., shooting from the closest boxes to the farthest ones.

All VR devices are dichoptic, and any software used for VR-based training displays scenes dichoptically treat the subject. The difference between the present software and those previously used and validated for vision training is that the stimuli used have been specifically designed to benefit the amblyopic eye, being based on a Gabor sinusoidal grating. Specifically, these patches are only shown in the amblyopic eye, whereas the other eye receives the scene without the Gabor stimuli. The scene objects, where the patches were positioned, were specifically designed to favor the integration of the monocular stimulus in a binocular environment. The scene was a totally immersive scene over 360°.

**Figure 2:**
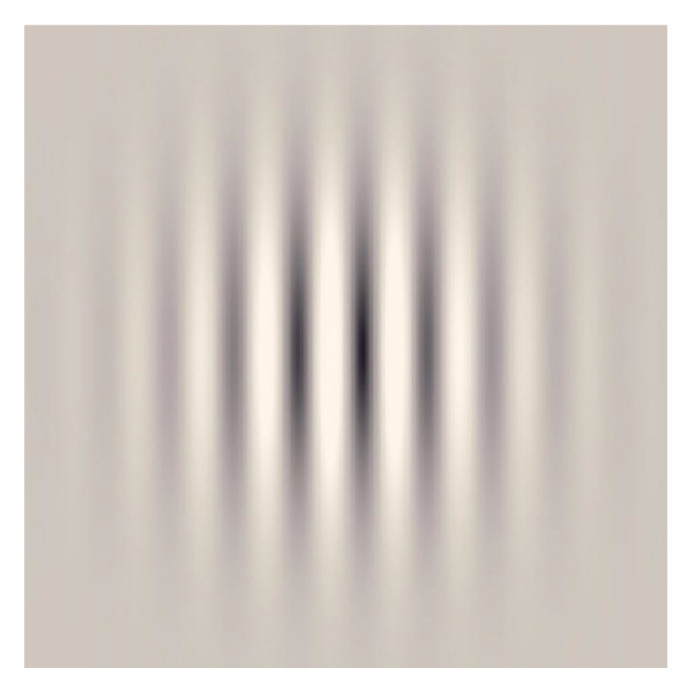
Gabor patches design used in the present study, allowing for luminance reproduction errors.

The Gabor patches were developed for different contrasts and calibrated in luminance according to a previous VR display characterization to ensure the correct contrast reproduction [25]. The characterization was performed experimentally using the CA-P427 Display Color Analyzer (Konica Minolta, Inc., Tokyo, Japan) and two 3DLUT tables, one for each eye/display [26]. The mean luminance of the pattern and background was configured at 50 cd/m^2^ according to our screen characterization. An example of this type of stimulus was represented in Figure 2. The maximum spatial frequency reproducible in a device is limited by the screen’s resolution and even more in the case of Gabor patches. Gabor patterns were created by modulating a sinusoidal grating with a Gaussian window, thus creating a soft transition between dark and soft patterns and consequently needing more pixels to create a cycle between patterns. The maximum spatial frequency theoretically reproducible in our screen was calculated to be 6 cycles per degree (cpd), but a stimulus of 3 cpd was finally defined to ensure the soft transition. Four possible orientations (0°, 45°, 90°, and 135°) were defined. The orientations were arbitrarily presented to observers, and changed over trials when a correct response was detected.

The contrast of the stimuli (difference in luminance between dark and soft patterns) was reduced during the training depending on the subject’s responses, decreasing the contrast of the stimuli when right responses were provided and increasing the contrast in the case of wrong answers. Thus, the subject responses were guided around the threshold. The psychophysical method used for the changes in contrast during the training followed the best PEST methodology [27].

### 2.3. VR Device

The VR device used for the training was the HTC Vive Pro Eye (HTC Corporation, California, USA). This device includes one headset, two controllers, and two base stations. The headset comprises a 3.5″ Dual OLED screen placed in front of the eyes, providing each eye with a screen of 1440 × 1600 pixels of resolution. Between the screen and the eyes, two adjustable oculars focus the image without an accommodative response. Since the screen is placed approximately 65 mm from the eyes, the power of the aspheric diffractive lenses is about +16 D. The minimum interpupillary distance available due to the size of the lenses (with no possibility of approximating the oculars more) was 61 mm.

This VR device was designed for adults, and its use was not recommended for children following the manufacturer’s indications, resulting in a device not fully adapted to children’s anatomy. In younger children, interpupillary distances can be smaller than 61 mm, inducing a prismatic effect that could be intolerable. Therefore, a limit of 5 mm of decentration (8 PD) was permitted, and the viability of fusion was checked before VR training on each subject. This requirement of a minimum interpupillary distance of 56 mm was established as an additional inclusion criterion in our study.

### 2.4. Study Protocol

Subjects were examined in three visits: before initiating the VR-based training (visit 0); during the training (visit 1, 9 sessions); and just after the final session of training (visit 2, 18 sessions). Training sessions consisted of playing the three mini-games described for 10 min each, leading to a total duration of 30 min. In the case of study visits (visit 0, 1, and 2), the total examination time was about 1 h. A total of 18 sessions were performed during one month and a half, with at least three sessions per week. Training program protocol was previously described [28].

### 2.5. Methodology

Primary outcomes were distance (4 m) and near (40 cm) VA measured with a logMAR chart based on C Landolt optotypes (the maximum VA measurable with our test was −0.10 logMAR), distance (3 m) contrast sensitivity (CS) measured with the CSV1000 test (Vector Vision, Greenvile, OH, USA), and near (40 cm) stereopsis measured with two random dot tests, the TNO red-green test (Lameris, Netherland), and Randot polarized test (Stereo Optical Company Inc., Chicago, IL, USA), with different second arc steps between plates, different random dot size, and different dissociation method.

Additionally, variables such as monocular accommodative facility with ± 2 D flipper, fusional range of vergences measured with prism bar for distance (6 m) and near vision (40 cm), heterophoria measured with the cover test for distance (6 m) and near vision (40 cm), and near point of convergence (NPC) with an accommodative stimulus (with a size two lines larger than that corresponding to the best near VA measured in the amblyopic eye) were also measured on each visit. Binocular measures were obtained prior to monocular measures in all cases.

Subjects’ symptomatology was assessed by the simulator sickness questionnaire (SSQ) [29] just after the training (on visit 17) to assess the possible impact of the use of the VR system and to identify any discomfort from subjects. This questionnaire comprises 16 items, such as fatigue, headache, dizziness, sweating, nausea, or blurred vision, among others, and every item was scored as 0 (never), 1 (mild), 2 (moderate), and 3 (severe) [29].

Data was collected and summarized in tables and figures, as shown in the Results section.

## 3. Results

All participants had moderate anisometropia, with a difference between fellow eyes in the objective refraction equivalent sphere from 2 to 3.5 D. Two subjects had mild to moderate amblyopia, with a difference of two lines (subjects 1 and 4) or three (subject 3) between eyes, whereas one subject had severe amblyopia with a difference of seven lines between eyes (subject 2). Two subjects were teenagers (subjects 1 and 4) and had been previously treated (patching and conventional vision therapy). Both had no treatments for years at the time of inclusion. The other two subjects were younger (subjects 2 and 3) and had been treated with patching for at least 1 year without improving vision in the last six months. Subjects’ demographics and baseline characteristics are summarized in Table 1.

Primary outcomes at baseline, intermediate, and final visits are summarized in Table 2. Distance and near VAs in dominant eyes were 0.00 logMAR or better in all cases. Distance VAs in amblyopic eyes remained stable in the two teenagers after the training, whereas the younger subjects improved 3 and 4 logMAR lines, respectively. Near VA remained constant in one subject, whereas it improved in the other three.

Stereopsis was measured with two tests, TNO and Randot, obtaining an acceptable correlation between tests. All subjects showed improved stereopsis of at least one step with both tests. Three subjects showed a final stereopsis of 60 s arc with both tests.

Accommodative and vergence characteristics of subjects included in this pilot study at baseline, intermediate, and final visits are summarized in Table 3. Accommodative facility showed asymmetries between eyes in three subjects, the amblyopic eye being the one that showed lower facility. The number of cycles per minute was slightly increased in the amblyopic eye after the training (also in the fellow eye), reducing the difference between eyes slightly. Only one subject showed no differences in the accommodative facility after training, but this subject had excellent baseline values; therefore, the possibility of improving was small. In the case of NPC, the post-training values showed an increase in the distance from eyes in one subject, a decrease in two subjects, and one remained constant. In the case of fusional vergences, the magnitude increased slightly in some cases, whereas in others decreased, with no clinically significant changes considering the measurement technique, prism bar with limited steps.

**Figure 3:**
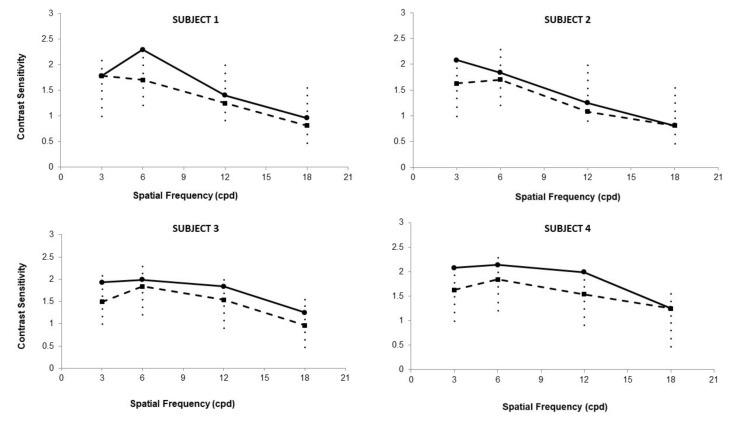
Contrast sensitivity of the amblyopic eye at baseline (discontinuous line) and final visit (continuous line) for 3, 6, 12, and 18 cpd (SUBJECT 1–4) obtained with the CSV1000 test. Small dots represent the possible values reportable depending on the spatial frequency evaluated, that is, the possible steps in the response of this test.

CS results for 3, 6, 12, and 18 cpd at baseline and final visit for the amblyopic eye are displayed in Figure 3. Three subjects (2, 3, and 4) showed a CS increase of approximately 0.5 CS units for the spatial frequency of 3 cpd after the training. Subject 1 also showed a similar increase for the spatial frequency of 6 cpd.

The results of the SSQ are summarized in Table 4. The most common self-reported symptom was the fullness of the head to a mild degree by all subjects. Fatigue, headache, and difficulty in focusing were also reported, to a mild degree, by half of the sample. One subject reported moderate fatigue and sweating, and another had moderate headache and eyestrain. This subject (subject 1) also reported severe difficulty in focusing.

All subjects performed the 18 sessions of 30 min without exception in the time required, and therefore compliance was complete.

## 4. Discussion

Dichoptic training using digital devices is based on reproducing different but complementary stimuli for each eye and controlling the information provided. Specifically, there are different modes of providing dichoptic stimulation. One is to show in the same window the information of both eyes superimposed, providing each eye with a partial image using green-red glasses, polarized, or others. Another is to create two complementary scenes in separate windows and to use a physical dissociation method in which one eye cannot see the scene the other eye sees (with the use of a septum). This last one has the advantage of providing two images absolutely independent of each eye, allowing a complete dissociation [30], without the presence of ghosting images that can decrease the quality of the scene and create undesired interferences between eyes.

In the case of head-mounted displays (HMD) used for binocular training, they are based on this second type of dissociation. This sometimes can lead to confusion related to the concept of VR, with some authors considering each device based on this type of dissociation method using HMD as VR. The use of dichoptic stimuli with different disparities to create the perception of depth (stereograms) can be considered virtual stimulation, as stimuli are reproduced on a screen, but this concept cannot be confused with VR. VR experience depends on the implication and participation of the observer in an immersive environment of 360° to create the perception of being in a real environment created virtually. This situation is not present when the subject’s task is to watch a dichoptic 3D movie using an HMD. Indeed, the only difference with other dichoptic training is the dissociation method, which uses an HMD instead of an external screen with chromatic or polarized glasses.

When analyzing the scientific evidence regarding the use of VR for vision training, it is important to know the device and software used and, consequently, the level of subject involvement to ensure that we are talking about real VR. Concerning previously published studies about the use of VR training on children with amblyopia [21,22,23], there were some doubts when analyzing the training software, with no clear identification as to whether the visualization was done in a real immersive VR environment or not. In the case of Elhusseiny et al. [21], the device used was the VR One Plus virtual reality headset (Zeiss, Germany), with an iPhone 6 plus smartphone preloaded with the therapeutic software, but no information about the scenes of this software was provided. These authors randomized participants in the treatment group and a sham-crossover group, both working with the same software. However, in the case of the study group, the stimulus of the fellow eye and amblyopic eye were altered during the visualization. In the case of Tang and Yang et al. [23], the authors clearly described the games used and defined the subject’s tasks in the two study groups, one using VR and the other using augmented reality (AR). In this study, although the stimuli were described in detail, it also remained unclear if the rest of the scene was an immersive background. In the case of Rajavi et al. [22], the authors used the I-BiT system, which is an adaptation of the well-described I-BiT™ system [31], combined with a software allowing to perform VR tasks. Unfortunately, no information about the hardware and the software of this new approach could be obtained, neither in research databases nor in commercial websites, to determine its fully immersive nature.

In the present pilot study, the software prototype displayed a real immersive VR scene in which the mini-games were integrated, allowing the subject the observation of the target stimuli but in a real binocular tri-dimensional environment over 360º. It is still unknown how fully immersive training could benefit the efficacy of training over using a non-immersive scene with an HMD, and future studies comparing immersive vs. not immersive training in amblyopia should be performed. The immersive nature of the software used is a differentiating element compared to others, but whether this fact is an advantage or not in terms of treatment efficacy should be investigated further. The present software prototype requires a high-performance computer (impossible with a smartphone) and a previously characterized VR screen for correct contrast reproduction.

The training was developed at an office under the supervision of an optometrist, contrasting with the use of headsets with integrated smartphones for home-based vision therapy [21,22]. This was an additional challenge for subject recruitment since participants must be available to attend 18 in-office training sessions. However, the great advantage was that the subject’s compliance was complete. Additionally, the attractiveness of a game in comparison to patching has demonstrated its advantages for compliance when used for home-based training [32]. In our case, although the treatment was office-based, the attractiveness of an immersive VR game, along with the previous failure of patching in some cases, were strong motivating factors for compliance.

### 4.1. Visual Acuity

VA measurement was one of the main outcomes of analyzing the training improvement in amblyopia. In the four cases evaluated, distance VA improvements were found in the amblyopic eye in two of the four subjects (50%) and near VA in three of the four subjects (75%). Improvements were of more than one line of VA measured with a logMAR scale and can be considered clinically relevant. These improvements were more marked in the two subjects of less than eight years old. A reason for these improvements could be the age of subjects since other authors have found differences in VA in younger children with dichoptic training [23]. Tang and Yang et al. [23] studied the effect of short-term visual performance in 71 anisometropic children with a mean age of six and found statistically significant differences in VA after the training. Other authors have studied the effects of long-term training and did not find significant differences in VA between children and adults, as in the case of Elhusseiny et al. [21]. In any case, it should be considered that these authors included in their sample subjects with unilateral amblyopia due to anisometropia and/or strabismus. On the other hand, in our mini sample, these two subjects with no improvements in VA had a higher baseline VA and, therefore, lower possibilities of improvement.

### 4.2. Contrast Sensitivity

Deficits in CS in amblyopic subjects are common, especially for medium and high spatial frequencies [2]. Therefore, the measurement of CS should be considered a crucial parameter in evaluating the visual improvement of the amblyopic eye. Training CS by perceptual learning tasks in amblyopic subjects has been demonstrated to be especially efficacious when using Gabor patches stimuli [24]. In the present study, the purpose was to integrate this type of stimuli in a VR environment to provide the amblyopic eye an additional stimulation. Indeed, all subjects showed improvements in CS, specifically at the frequency of 3 cpd in three subjects, which was the frequency employed during the training sessions. These improvements were also present in the remaining spatial frequencies, 6, 12, and 18 cpd. Other authors, such as Tang and Yang et al., also studied changes in CS after the short-term training with integrated Gabor patches [23] and found significant improvements in CS for the spatial frequency of 12 cpd. Unfortunately, the authors did not report the spatial frequencies used in their Gabor patch stimuli during the training. Future studies should investigate how the training of specific spatial frequencies could affect the improvements in CS. However, nowadays, the spatial resolution of the VR displays is still a limitation for reproducing high spatial frequencies [33], especially if a stimulus with a sinusoidal contrast transition and a Gaussian filter, such as the Gabor patches, is intended to be displayed.

### 4.3. Stereopsis

Stereoscopic vision is the most beneficial of binocular treatments [2]. In the case of VR, dichoptic training is guaranteed, with some authors demonstrating improvements in stereoscopic vision after VR-based training in adults with amblyopia [17,18,19,20]. In the case of amblyopic children, some authors have studied the short-term [23] and long-term [21] results of vision training. As mentioned before, Elhusseiny et al. [21] studied a sample of 20 older children and adults that were randomized into a treatment group and a sham cross-over group. They found significant improvements for the entire cohort after 8 weeks of training in stereoacuity measured with the Titmus Fly test. In fact, both the study and control cross-over groups performed a binocular task with the same device and software. Tang and Yang et al. [23] studied a sample of 145 subjects randomized in a VR group and AR group and trained for 20 min, and also found significant improvements in stereopsis in both groups measured with a random dot test.

### 4.4. Limitations

One of the main limitations found for the inclusion of participants was the difficulty in finding older children with amblyopia due to anisometropia, since visual screening campaigns and early detection of refractive problems in the pediatric population, along with the appropriate prescription of patching and vision therapy treatments, have decreased the prevalence of anisometropic amblyopia in our clinics. In the case of younger children, the problem was to find participants with an IPD of more than 56 mm. As previously mentioned, determining IPD is essential for adequate training performance since a minimum decentration of the positive lenses from the pupillary center of the eye could lead to considerable prismatic effects. Considering Prentice’s rule, a temporal decentration of the lenses (VR with a separation between oculars greater than the subject’s IPD) will induce a temporal base effect of 1.6 PD for 1 mm, 8 PD for 5 mm, or even 16 PD for 10 mm of decentration. The real prismatic effect of the lenses cannot be obtained from the VR device (lenses cannot be separated from the screen), and therefore only theoretical calculations can be done. The adjustment of the IPD of subjects is mandatory when using a VR device, and even more so if these devices are used for training purposes. In our case, IPD was an additional inclusion criterion that limited the participation of some subjects of younger ages.

In addition to the small sample size evaluated, another limitation of the study was the variability of some binocular vision measurements with some techniques, such as measuring vergences with a prism bar, measured only once during each visit. Furthermore, several of these techniques, such as measuring the accommodative facility, fully depended on the children’s subjective responses. CS measurement with the CSV1000 test also has the inconvenience that there are only two response options for each frequency, and therefore, a correct guessing rate of 50% can be assumed. For this reason, binocular results should be considered cautiously, although no clinically relevant deterioration of binocular parameters was observed. Only the accommodative facility experienced improvements in our amblyopic children with training that should be investigated further in future studies. In any case, changes in the accommodative facility have also been reported by other authors in normal subjects [34,35] that align with our findings.

## 5. Conclusions

Results from this pilot study suggest that visual training based on perceptual learning through an immersive VR environment could be a viable treatment for improving CS, VA, and stereopsis in some children with anisometropic amblyopia. Future studies should be conducted with large subject samples to validate these preliminary results.

## Figures and Tables

**Table 1:** Summary of demographics and baseline (visit 0) characteristics of subjects included in this pilot study. Abbreviations: IPD, interpupillary distance; X, exo; E, eso; F, phoria; T, tropia; * RE suppression from 1 m in advance.

Subject	Age (Years)	Gender (Male/Female)	IPD (mm)	Refraction (Diopters)	Cover Test (Prismatic Diopters)	Worth Dot Test	Stereopsis (Seconds of Arc)
1	14	F	66	RE +0.75 −0.50 180	Distance 2 XF	Distance 4	TNO 120
				LE +4.25 −1.00 180	Near 8 XF′	Near 4	Randot 200
2	8	F	57	RE +4.50 −1.50 180	Distance 2ET	Distance 4	TNO 480
				LE +1.50 −0.75 110	Near 4 XF′	Near 4 *	Randot 400
3	8	F	62	RE +3.25	Distance 0	Distance 4	TNO 120
				LE +0.00	Near 4XF′	Near 4	Randot 100
4	14	M	58	RE +5.25	Distance 0	Distance 4	TNO 240
				LE +8.00 −1.25 160	Near 2XF′	Near 4	Randot 200

**Table 2:** Primary outcomes (VA and Stereopsis) in all subjects at baseline (visit 0), intermediate (visit 1), and final (visit 2) visits. Abbreviations: VA, visual acuity; ∆VA, the difference of visual acuity between pre- and post-training in the amblyopic eye.

Subject		Distance VA		Near VA		Stereopsis	
		Dominant	Amblyopic	Dominant	Amblyopic	TNO	Randot
1	Visit 0	−0.10	0.20	−0.10	0.20	120	200
	Visit 1	−0.10	0.28	−0.10	0.10	120	100
	Visit 2	−0.10	0.20	−0.10	0.20	60	60
∆ VA			+0.00		+0.00		
2	Visit 0	−0.10	0.62	+0.00	0.30	480	400
	Visit 1	−0.10	0.32	−0.10	0.20	240	400
	Visit 2	−0.10	0.22	−0.10	0.10	240	200
∆ VA			+0.40		+0.20		
3	Visit 0	0.02	0.34	−0.10	0.00	120	100
	Visit 1	−0.10	0.14	−0.10	−0.10	240	100
	Visit 2	−0.08	0.02	−0.10	−0.10	60	60
∆ VA			+0.32		+0.10		
4	Visit 0	−0.08	0.22	−0.10	0.40	240	200
	Visit 1	−0.10	0.22	−0.10	0.10	60	60
	Visit 2	−0.06	0.24	−0.10	0.00	60	60
∆ VA			−0.02		+0.40		

**Table 3:** Accommodative and vergences characteristics of subjects included in this pilot study at baseline (visit 0), intermediate (visit 1), and final (visit 2) visits. Abbreviations: FAM, Facility of Accommodation in monocular conditions in cycles per minute (cpm); NPC, near the point of convergence; NFV, negative fusional vergences measured in PD, prismatic diopters; PFV, positive fusional vergences. Only break and recovery of fusional vergences were recorded.

Subject		FAM (cpm)		NPC (cm)	NFV (PD) (Break/Recovery)		PFV (PD) (Break/Recovery)	
		Dominant	Amblyopic		Distance	Near	Distance	Near
1	Visit 0	10	3	4	8/6	20/16	16/8	14/10
	Visit 1	10	3	4	8/6	25/18	16/8	16/10
	Visit 2	14	6	9	8/6	25/18	12/8	14/8
2	Visit 0	7	1	15	8/4	16/12	12/10	10/4
	Visit 1	7	9	13	4/2	16/12	8/4	8/6
	Visit 2	-	-	14	6/4	16/12	10/8	8/6
3	Visit 0	13	9	10	10/8	16/10	20/16	25/20
	Visit 1	10	16	7	10/8	18/16	20/10	18/16
	Visit 2	16	11	7	8/6	25/14	16/10	25/18
4	Visit 0	14	14	10	8/6	25/16	8/6	30/25
	Visit 1	18	16	4	6/4	8/4	25/18	30/25
	Visit 2	16	16	3	6/4	14/10	20/10	25/20

**Table 4:** Summary of SSQ responses after one session of training (session 17) for subject 1 (†), subject 2 ($), subjects 3 (&), and subject 4 (@) for every evaluated item.

	Never	Mild	Moderate	Severe
General Sickness	† $ &	@		
Fatigue	@	† $	&	
Headache	&	$ @	†	
Eyestrain	$ & @		†	
Difficulty Focusing	&	$ @		†
Salivation Increasing	† $ @	&		
Sweating	† $ @		&	
Nausea	† $ & @			
Difficulty Concentrating	† $ & @			
Fullness of the Head		† $ & @		
Blurred Vision	† $ & @			
Dizziness with Eyes Open	† $ @	&		
Dizziness with Eyes Closed	† & @	$		
Vertigo	† $ & @			
Stomach Awareness	† & @	$		
Burping	† $ & @			

## Data Availability

Data is contained within the article.
